# Large area and deep sub-wavelength interference lithography employing odd surface plasmon modes

**DOI:** 10.1038/srep30450

**Published:** 2016-07-28

**Authors:** Liqin Liu, Yunfei Luo, Zeyu Zhao, Wei Zhang, Guohan Gao, Bo Zeng, Changtao Wang, Xiangang Luo

**Affiliations:** 1 State Key Laboratory of Optical Technologies on Nano-Fabrication and Micro-Engineering, Institute of Optics and Electronics, Chinese Academy of Science, P.O. Box 350, Chengdu 610209, China

## Abstract

In this paper, large area and deep sub-wavelength interference patterns are realized experimentally by using odd surface plasmon modes in the metal/insulator/metal structure. Theoretical investigation shows that the odd modes possesses much higher transversal wave vector and great inhibition of tangential electric field components, facilitating surface plasmon interference fringes with high resolution and contrast in the measure of electric field intensity. Interference resist patterns with 45 nm (∼*λ*/8) half-pitch, 50 nm depth, and area size up to 20 mm × 20 mm were obtained by using 20 nm Al/50 nm photo resist/50 nm Al films with greatly reduced surface roughness and 180 nm pitch exciting grating fabricated with conventional laser interference lithography. Much deeper resolution down to 19.5 nm is also feasible by decreasing the thickness of PR. Considering that no requirement of expensive EBL or FIB tools are employed, it provides a cost-effective way for large area and nano-scale fabrication.

With the rapid development of nanotechnology, large area and nano-scale fabrications are being massively and increasingly demanded. As an effective tool for producing nano patterns, laser interference lithography possesses the advantages of low cost, large area, and no need of precise focusing^1^, and shows promising applications in manufacturing variant functional devices, such as grating, arrayed patterns for light harvesting^2^, two dimensional and three dimensional photonic crystal structures^3^,^4^ etc. The half pitch resolution of conventional interference lithography, however, is theoretically limited by one quarter of light wavelength. For the improvement of resolution, light sources with shorter wavelength have been demonstrated, such as deep ultraviolet (DUV)^5^, extreme ultraviolet (EUV)^6^,^7^, and soft X-ray^8^. Unfortunately, the expensive laser setups and complex processing impede their practical applications.

Recently, a variety of works demonstrated that surface plasmon polaritons (SPPs) with much shorter propagation wavelength than that in vacuum delivers a potential access to break the resolution limit of interference lithography^9^,^10^,^11^,^12^. For instance, silver grating with 50 nm slit width and 300 nm pitch was employed to achieve SPP modes interference at the wavelength of 436 nm, obtaining about 100 nm pitch resist patterns (PR)^10^. It was also demonstrated that SPPs help to amplify evanescent waves and realize super-resolution imaging lithography as well^13^,^14^,^15^,^16^. Whereas, the critical dimension of grating structures used to excite SPPs is always in the deep sub-wavelength scale, and expensive tools like electron beam lithography (EBL) and focused ion beam (FIB) are usually employed in SPP interference lithography investigations^9^,^10^,^17^,^18^,^19^. To relieve this problem, prism with Kretschmann or Otto schemes could also excite SPPs and realize large area and maskless interference lithography^20^, but its resolution is restricted by the refractive index of prism materials. Recently, bulk plasmon polaritons (BPPs) structure with hyperbolic metamaterials composed of multiple metal and dielectric films were successfully used to realize 45 nm half-pitch interference patterns^21^. As the cost of its unique ability to realize much smaller interference period than that of excitation grating, even with demagnification factor of 4 times, the low light efficiency inside multiple metal films is not welcome in applications.

In this paper, we demonstrated large area, uniform, high contrast, and high aspect-profile deep sub-wavelength interference lithography by exciting odd SPP modes in metal/insulator/metal (MIM) structure. The unique features of odd SPP modes, including shorter propagation wavelength, inhibition of tangential electric field components and light intensity enhancement^12^, are engineered for deep sub-wavelength SPP lithography. In addition, the grating structure used to excite SSPs could be readily fabricated by simple and low-cost conventional laser interference lithography. As an experimental demonstration, interference patterns with half pitch 45 nm (~*λ*/8), 50 nm depth, and area size up to 20 mm × 20 mm were presented. Furthermore, some discussions like the methods to improve resolution and surface roughness influence are presented as well.

## Results

### Principle and design of odd SPP modes interference structure

**Figure 1:**
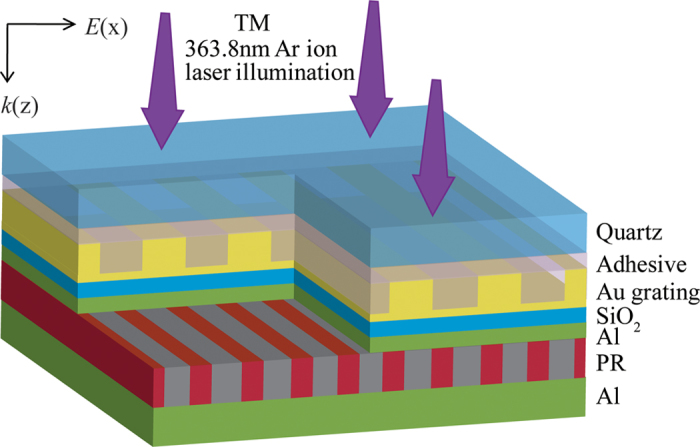
Schematic of odd SPP modes interference structure.

The schematic of odd SPP modes interference structure is presented in Fig. 1. A plane wave of 363.8 nm wavelength and in transverse magnetic (TM) polarization, normally impinges Au grating with 180 nm pitch and 40 nm deep grooves. Below it are 20 nm-thick SiO_2_ layer and 20 nm-thick Al/50 nm-thick PR/50 nm-thick Al structure, where deep sub-wavelength interference patterns are recorded by the sandwiched PR layer. The relative permittivity of Au, SiO_2_, Al, PR are −0.429 + 5.73i, 2.13, −19.42 + 3.60i, and 2.56 at light wavelength of 363.8 nm^16^,^21^,^22^, respectively. And all the materials are assumed to be nonmagnetic with permeability *μ* = 1.

**Figure 2:**
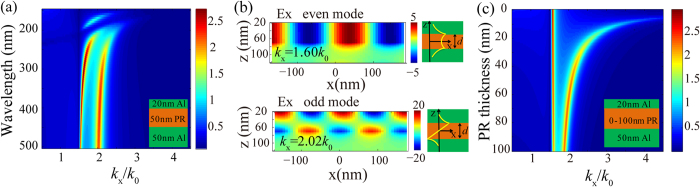
(**a**) Calculated magnetic field amplitude inside Al/PR/Al structure as a function of wavelength and normalized transverse wave *k*_x_ for 50 nm-thick PR. (**b**) The electric field distributions of even and odd SPP modes for *k*_x_ = 1.60*k*_0_ and 2.02*k*_0_ respectively, and the two schematic diagrams show electric field profiles for the two modes. (**c**) Calculated magnetic field amplitude in the middle plane of Al/PR/Al structure for variant PR thickness and transverse wave *k*_x_ and fixed light wavelength 363.8 nm.

To show the SPP modes behavior inside the MIM structure, Fig. 2 presents the calculated results of light field inside with propagation matrix method, by scanning plane wave light incidence with variant transversal wavevector *k*_*x*_ impinging 20 nm Al/ PR /50 nm Al waveguide structure. In the case of fixed PR thickness 50 nm, Fig. 2(a) gives calculated magnetic field *H*_*y*_ amplitude inside MIM as a function of wavelength and transverse wave *k*_*x*_. Obviously, as the incident light matches the waveguide modes inside, great magnitude of light field could be observed. Two SPP modes are excited and positioned on the right side of the light line *k*_*x*_ = *k*_0_. According to the waveguide theory^23^,^24^,^25^,^26^,^27^, one is even SPP modes with relative low *k*_*x*_, and the other is odd modes with much higher *k*_*x*_. This point could be further seen from the calculated *E*_*x*_ field distributions in Fig. 2(b), corresponding to the two SPP modes at *k*_*x*_ = 1.60*k*_0_ and 2.02*k*_0_ respectively for light wavelength 363.8 nm. The former one shows nearly symmetrical *E*_*x*_ field profile with respect to the middle line of PR layer, and the latter anti-symmetrical profile. In approximation to an ideal structure with infinite thickness of both two Al films, their dispersion relations could be defined as


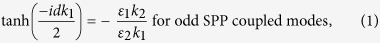



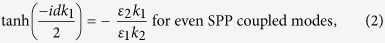


where *ε* represents the dielectric constant, and the subscripts 1 and 2 indicate the PR and Al; *k* is defined by the momentum conservation 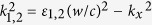; *d* is the thickness of PR.

The two modes show different dependence on the variation of PR thickness, as demonstrated in Fig. 2(c) with 0 nm~100 nm thick PR and fixed light wavelength 363.8 nm. The even SPP modes are much close to the light line and keep nearly fixed for variant PR thickness. This resembles the case of the no cut-off plane wave modes propagating inside a gap between two semi-infinite perfect conductors. On the other hand, the odd modes are strongly affected by the PR thickness, and *k*_*x*_ increases greatly as the PR thickness decreases. So the resolution of the interference patterns could be improved by optimizing the thickness of PR. As the demonstrative example for odd SPP mode interference lithography, the normal light incidence with wavelength 363.8 nm and grating period 180 nm correspond to odd SPP modes in 20 nm Al/50 nm PR/50 nm Al structure with *k*_*x*_ = 2.02*k*_0_.

**Figure 3:**
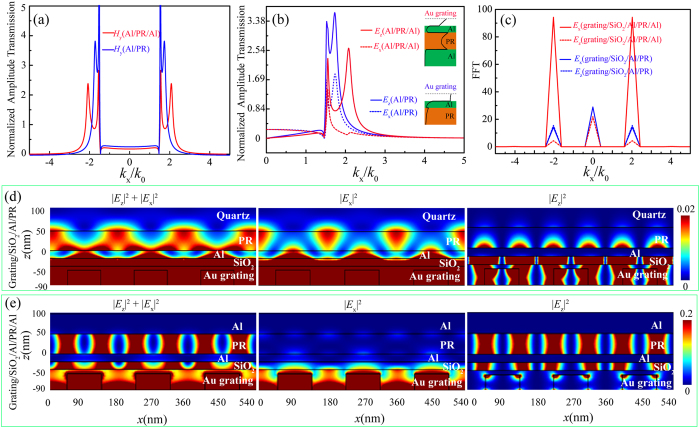
For Al/PR and Al/PR/Al structures, normalized amplitude transmission in the measure of |*H*_y_| in (**a**), |*E*_z_| and |*E*_x_| in (**b**). The two insets in (**b**) are electric field intensity distributions for odd and even SPP modes. (**c**) The Fourier spectra of interference fringes with |*E*_z_| and |*E*_x_|, (**d**) and (**e**) are |*E*_z_|^2^ + |*E*_x_|^2^, |*E*_x_|^2^, and |*E*_z_|^2^ light distributions, for grating/SiO_2_/Al/PR and grating/SiO_2_/Al/PR/Al structures, respectively.

The odd SPP modes exhibit unique features of electric field components profiles, in comparison with the SPP modes just with Al/PR structure. To address this point, Fig. 3(a,b) present the calculated |*H*_*y*_|, |*E*_*z*_| and |*E*_*x*_| transmission amplitude for the two structures, namely 20 nm Al/50 nm PR and 20 nm Al/50 nm PR/50 nm Al. The input and observation planes here are fixed at the top Al film surface and the middle of PR layer, and the contribution of bottom Al film is considered here as well in the calculation. Figure 3(a) shows that Al/PR/Al structure delivers odd SPP modes with much higher propagation wavevectors than that of SPP modes in Al/PR structure. Figure 3(b) exhibits the great different amplitude transmission profiles in the measure of |*E*_*z*_| and |*E*_*x*_| for Al/PR/Al structure. The |*E*_*x*_| amplitude transmission shows great inhibition around the odd mode position, with magnitude much smaller than |*E*_*z*_| amplitude transmission values. And no obvious difference of amplitude transmission profiles lies between |*E*_*x*_| and |*E*_*z*_| for Al/PR structure. This phenomenon occurs mainly due to the strong SPP coupling between the top and bottom Al surfaces facing the PR layer.

To ensure high contrast and uniform interference fringes, it is commonly desired that two SPP modes with opposite propagation directions, equal magnitude and proper electric field components profiles exist in the PR layer. In our design, the grating takes the period of 180 nm and is illuminated normally by the laser beam, for the sake of generating two symmetrical odd SPP modes. Clearly, those diffraction light of grating with transverse wavevectors matching the odd SPP modes would be enhanced and dominate the light behavior in PR layer. This occurs for the Al/PR/Al structure as shown in Fig. 3(a), where the ±1 nd orders with ±2.02*k*_0_ nearly coincide with the peak position of odd SPP modes. At the same time, the other light diffraction orders, like the 0th and ±2nd orders at 0 and ±4.0*k*_0_, would be suppressed greatly as could be also seen from the Fourier spectrum analysis shown in Fig. 3(c). For the Al/PR structure, however, the SPP modes are not resonantly excited and the 0th light diffraction with considerably high magnitude at *k*_*x*_ = 0 appears in PR layer.

The amplitude transmission difference between |*E*_*z*_| and |*E*_*x*_| for odd SPP modes in MIM structure plays the key role for high contrast interference fringes in the measure of |*E*_*z*_|^2^ + |*E*_*x*_|^2^. For Al/PR structure, Fig. 3(b) shows the |*E*_*z*_| and |*E*_*x*_| have nearly the same amplitude at the 2.02*k*_0_. And greatly blurred interference fringes of |*E*_*z*_|^2^ + |*E*_*x*_|^2^ are generated due to the fringe shift between |*E*_*z*_|^2^ and |*E*_*x*_|^2^ profiles, arising from the *π*/2 phase difference between them, as shown in Fig. 3(d). Also the great magnitude of 0th diffraction order would contribute negatively to the contrast of fringes in this case. Benefiting from the inhibition of |*E*_*x*_| components in Al/PR/Al structure for the odd SPP modes, with magnitude ratio |*E*_*z*_|/|*E*_*x*_| being 21.43 at 2.02*k*_0_ shown in Fig. 3(b), about 20 times that of Al/PR structure, interference fringe of |*E*_*z*_|^2^ + |*E*_*x*_|^2^ in Fig. 3(e) exhibits high contrast and uniform patterns with period here being 0.5 times that of excitation grating. In addition, the fringe intensity here is about 25.57% times that of incident light, about 20 times that of Al/PR structure.

**Figure 4:**
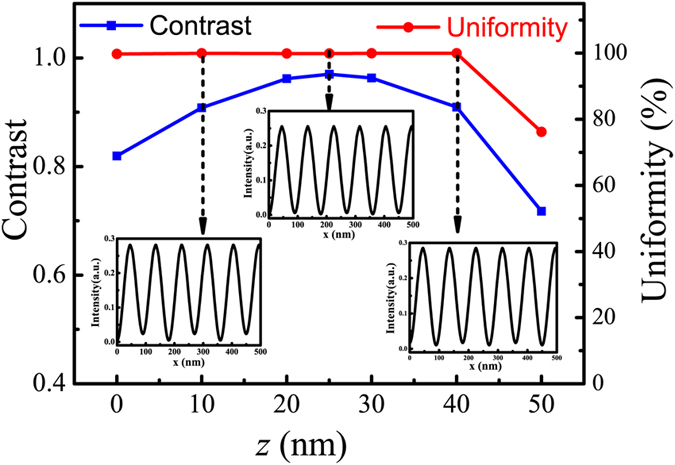
Contrast and uniformity of interference fringes at variant positions in PR layer and insets show the fringe profiles at three positions.

To further demonstrate the property of the calculated odd SPP modes interference fringes, Fig. 4 presents the value of contrast and uniformity at variant depth position inside PR layer. Insets from left to right are interference light intensity distribution corresponding to PR depth 10 nm, 25 nm, and 40 nm, respectively. The calculated average contrast is usually larger than 0.9, except for those at the two sides of PR layer close to Al films, where some distortions exists due to the not well damped |*E*_*x*_| components. Moreover, average uniformity of interference fringes is 96.49%. The calculating methods of intensity efficiency of light field, contrast, and uniformity are given in the Method part.

### Experimental results

**Figure 5:**
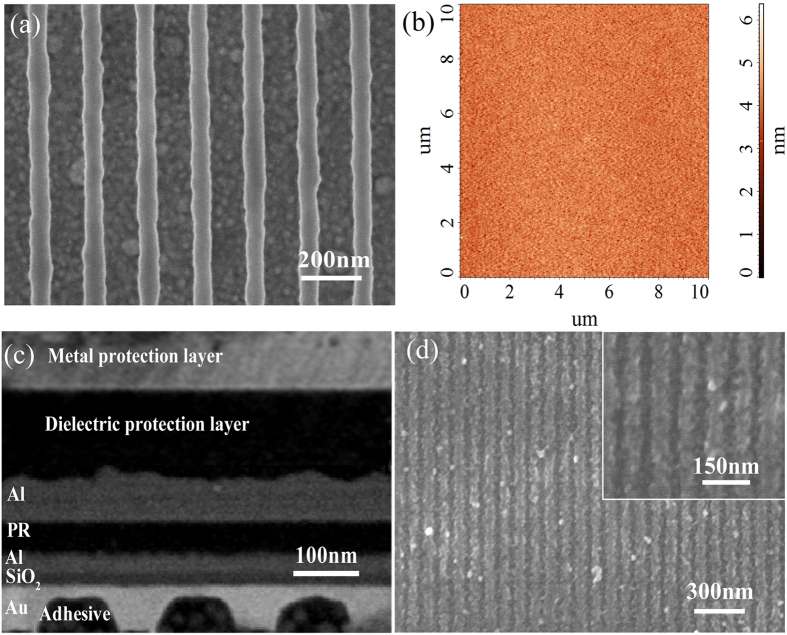
(**a**) SEM picture of Au grating SPP modes excitation. (**b**) AFM measured surface morphology of Au grating after TS processing, RMS 0.4 nm. (**c**) Cross section picture of SPP interference structure. (**d**) Odd SPP modes interference lithography patterns with 45 nm half pitch.

Before interference lithography performance, the structure with grating and MIM, as depicted in Fig. 1, was specially fabricated with details shown in Methods section. One key point is that the surface roughness of the films structure, especially for the Al film, need to be carefully controlled to obtain high contrast and uniform interference fringes. First, a 180 nm-pitch Au gratings, as shown in Fig. 5(a),was successfully fabricated on a Si wafer, by etching PR patterns produced with immersion interference lithography. Then the Au gratings were transferred from Si wafer to SiO_2_ substrate by template stripping (TS) method^28^,^29^,^30^, producing a planar profile with the morphology and smoothness inherited from the smooth Si (100) wafer. As shown in Fig. 5(b), the measured root mean square (RMS) roughness is about 0.4 nm in AFM scan area 10 μm × 10 μm. After that, the multilayer films with 20 nm SiO_2_/20 nm Al/50 nm PR/50 nm Al were fabricated on this TS substrate respectively, corresponding to roughness RMS 0.7 nm/0.9 nm/0.4 nm for the first three films (Fig. S1). It should noted that the 20 nm Al film has 1.8 nm RMS in the case of pure Al as the sputter target. Here alloyed Al sputter target with 3% Cu^31^,^32^,^33^,^34^ was employed to reduce surface roughness.

Figure 5(c) presents the cross-sectional SEM image of the sample for odd SPP interference lithography experiment. The top two layers in Fig. 5(c) are metal and dielectric protective layers to avoid sample damage during cross-section milling. Figure 5(d) demonstrates the clearly resolved and uniform PR grating lines with 45 nm half pitch (~*λ*/8) produced by odd SPP interference lithography. The fringe lines exhibit rough line edge, mainly caused by the limited resolution of i-line resist and roughness of films. The size of interference pattern region is about 20 mm × 20 mm, which is almost the same as that of Au grating. The experimental results are consistent with that of simulation in Fig. 3(e), demonstrating that odd SPP modes are successfully excited to generate deep sub-wavelength and large-area interference patterns. As the odd SPP interference pattern holds good contrast and intensity inside the MIM structure (Fig. 4), the obtained interference pattern depth is about 50 nm, the same as resist thickness and much deeper than some plasmonic lithography without MIM structure^15^,^35^,^36^,^37^.

### Discussion of fringe quality and resolution improvement

**Figure 6:**
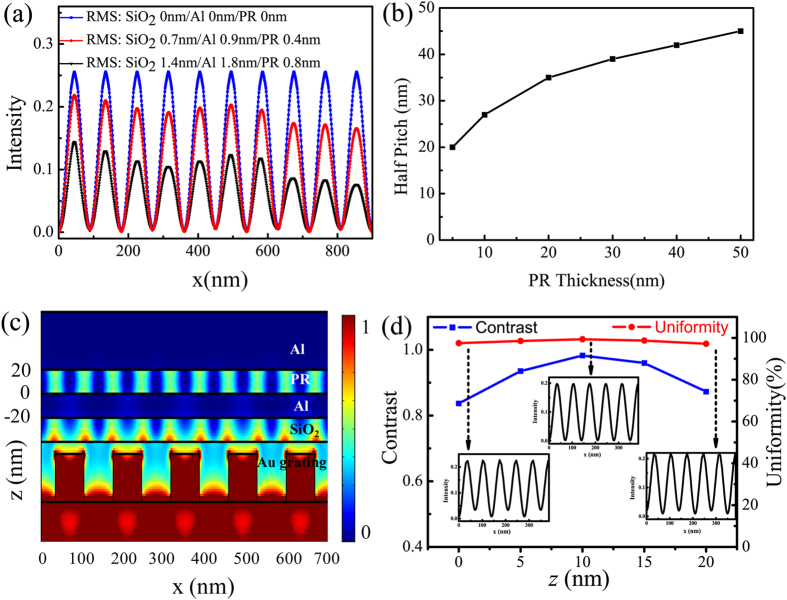
(**a**) Simulated odd SPP interference distribution in the middle plane of PR for structure with rough films. (**b**) The hp resolution of the odd SPP interference lithography increases as the PR thickness decreases. (**c**) Electric field intensity distribution in logarithm scale inside the odd SPP interference structure with mask pitch 140 nm and 20 nm-thick PR. (**d**) Contrast and uniformity of interference fringes at variant positions in PR layer and insets show the fringe profiles at three positions. The other parameters are the same as Fig. 1.

To demonstrate the negative influence of film roughness, Fig. 6(a) shows the simulated odd SPP interference distribution in the middle of PR, where solid square data represents smooth films with RMS 0 nm; solid circle data stands for experimental 20 nm SiO_2_/20 nm Al/50 nm PR films with RMS 0.7 nm/0.9 nm/0.4 nm respectively; solid triangle data with 2 times RMS of that experimental data. It is apparent that the film roughness delivers greatly reduced pattern uniformity to 72.50% and 37.12% in the last two roughness cases, respectively. Also reduced is the light intensity, of which the calculated normalized value is 22.57%, 19.24%, and 10.08% in the three cases respectively. This occurs mainly due to the light scattering on rough films with random light distribution and increase of energy loss. To further relax this concern, some advanced film deposition methods, such as molecular beam epitaxy (MBE)^38^, and optimization of films material parameters would be further exploited. For instance, some investigations show that appropriate light loss inside metallic films help to inhibit scattered light and bring much more uniform fringes^39^.

It is accessible to improve SPP interference lithography resolution by adjusting light wavelength and geometrical parameters, according to the investigation of SPP modes dispersion behavior exhibited in Fig. 2(a,c). With the decrease of PR thickness, the half pitch resolution of the odd SPP interference lithography is improved gradually, as illustrated in Fig. 6(b). Here, an example with 20 nm-thick PR is given for interference pitch shrinkage. As shown in Fig. 6(c), 35 nm (~*λ*/10) half pitch interference patterns are clearly presented in PR layer, corresponding to the odd SPP modes at about 2.60*k*_0_. Contrast and uniformity of the interference patterns are demonstrated in Fig. 6(d), average contrast 0.91, uniformity 98.23%, and normalized light intensity 19.74%. The half pitch could reach 19.5 nm (~*λ*/19) for 5 nm-thick PR and the simulated results are presented in Fig. S3. Utilization of light source with short wavelength is another access for resolution improvement. For instance, 15 nm half pitch SPPs interference is designed with 193 nm light wavelength^18^.

## Discussion

In summary, both the simulated and experimental results demonstrate that odd SPP modes with MIM structure could deliver large-area, high aspect-profile, and deep sub-wavelength interference lithography patterns. MIM structure could remarkably enhance the interference contrast, uniformity and intensity as well, provided that light wavelength and grating period are optimized to match the odd SPP modes. As experimental examples, 45 nm half pitch interference patterns with area 20 mm × 20 mm was obtained in 50 nm-thick PR layer. In addition, TS method and Al alloyed with 3% Cu sputtering deposition were employed to decrease the negative effect from film roughness. It is also demonstrated that higher resolution of odd SPP modes interference are also feasible by decreasing the thickness of PR. Since the excitation grating pitch is 2 times of that interference fringe, its fabrication is performed by simple and low-cost conventional laser interference lithography, without the need of expensive and low yield FIB or EBL. For the convenience of experiment, the interference resist patterns in this paper are fabricated upon the complex structure even with SPP excitation grating. It could be applied in many ways. For instance, one could use it as the imprinting template for further transfer. The interference lithography could also be performed in near field proximity performance with excitation grating and MIM structure on two separated substrates. This point was numerically demonstrated by inserting an air spacer between grating and MIM, as shown in Fig. S2. In this case, the subwavelength patterns could be readily transferred to functional layers below MIM.

## Methods

### Numerical simulation

Rigorous coupled wave analysis (RCWA) was employed to calculate the *H*-field transmission factor and even and odd electric field distribution in Fig. 2. The RCWA code was written based on the equations in ref. 40. The electric field intensity distributions shown in Figs 3 and 6, Figs S2 and S3 were simulated using the commercially available CST software.

### Calculating methods of contrast, uniformity and intensity efficiency of light field

For each interference pitch, contrast *V* can be calculated according to equation: 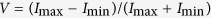
Where *I*_max_ and *I*_min_ represent the maximum and minimum intensity respectively. Contrast *V*_plane_ is defined as arithmetic mean value of all *V* in the same cross section plane. Average contrast is defined as arithmetic mean value of all *V*_plane_ in PR layer. Uniformity (*U*) is defined as 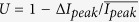, where 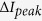 represents the maximum difference among the peaks, *I*_peak_ represents the mean light intensity peak of interference patterns. The average uniformity is the mean *U* in different depth PR. Intensity efficiency of light field according to the normalized light intensity.

### Structure fabrication

**Figure 7:**
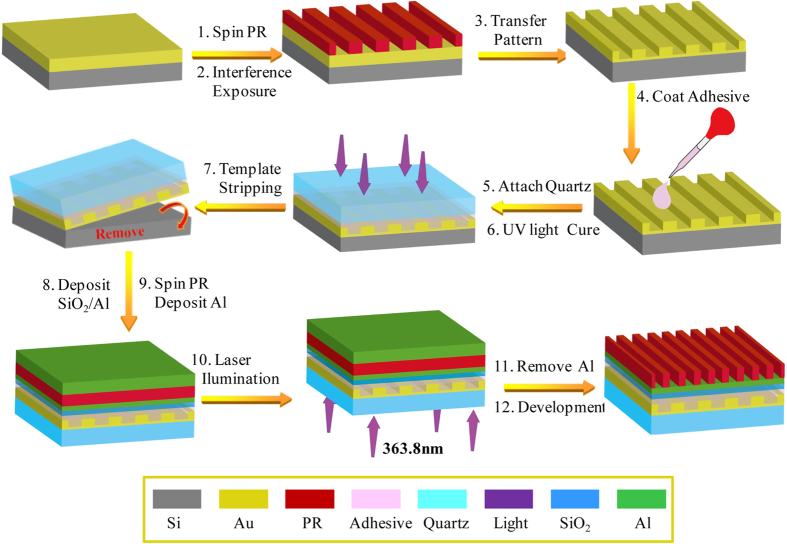
Schematic diagrams for the procedures of odd SPP interference structure fabrication and lithography.

Figure 7 depicts the procedures of structure fabrication and lithography process. The 50 nm-thick Au film was first deposited on Si (100) substrate by vacuum thermal evaporation at a base pressure 5.0 × 10^−4 ^Pa and deposition rate 0.5 nm/s. The 100 nm-thick AR-3170 positive PR (All-Resist GmbH, Strausberg) was spin-coated on the Au film, and then was baked at 100 °C for 10 min to drive off the residual solvent. Employing immersed interference lithography with 363.8 nm Ar-ion laser (Beam Lock 2060, Spectra-Physics), one-dimensional PR grating with 180 nm-pitch and about 20 mm × 20 mm area size was produced. Afterwards, the PR pattern was transferred into the Au film by ion beam etch (IBE). One should note that about 10 nm-thick bottom Au film was not etched as planarization layer for the next template stripping (TS) process. Using TS process, Au grating was transferred to quartz substrate from Si substrate with the help of UV curable epoxy resin adhesive (X31213 produced by ZYMET) that was cured in UV light illumination with energy density about 100 J/cm^2^. After that, 20 nm-thick SiO_2_ and 20 nm-thick Al films were deposited on this TS substrate by magnetron sputtering (DE500, DE Technology Limited) respectively, and it should note that sputter target for Al film is alloyed Al with 3% Cu. The diluted AR-3170 positive PR with thickness of about 50 nm was spin-coated on this Al film. After prebaking the PR for 5 min at 100 °C, 50 nm-thick Al film was deposited on the PR layer by vacuum evaporation at a base pressure 5.0 × 10^−4 ^Pa and deposition rate 2 nm/s.

### Lithography process

The PR in this structure was exposed by Ar-ion laser (TM polarized) with wavelength of 363.8 nm and exposure dose about 400 mJ/cm^2^. Before developing process, the 3 M adhesive tape was used to peel off the 50 nm-thick bottom Al film. After that, the exposed PR was developed for 14 s in a diluted solution of AR 300-35 (All-Resist GmbH, Strausberg) with volume ratio of 1:1 ratio by water, then it was rinsed with deionized water for about 10 s and dried by nitrogen respectively.

### Patterns measurement

The PR thickness was measured by surface profiler (Alpha step IQ_3_, KLA-Tencor). Grating patterns and sample profile were measured by scan electronic microscope (SEM) (SU8010, Hitachi). Sample profile was fabricated by milling ion milling system (IM4000, Hitachi). Film surface roughness was measured by atomic force microscope (AFM) (NT-MDT NTEGRA Spectra).

## Additional Information

**How to cite this article**: Liu, L. *et al*. Large area and deep sub-wavelength interference lithography employing odd surface plasmon modes. *Sci. Rep.* **6**, 30450; doi: 10.1038/srep30450 (2016).

## Supplementary Material

Supplementary Information
