# Supplementary material for: Large area and deep sub-wavelength interference lithography employing odd surface plasmon modes
Source: Sci Rep. 2016 Jul 28;6:30450. doi: 10.1038/srep30450 (PMC4964334; doi:10.1038/srep30450)
Supplement: Supplementary Information [file srep30450-s1.pdf]

Supporting Information

Large area deep sub-wavelength interference lithography employing odd surface plasmon modes

Liqin Liu, Yunfei Luo, Zeyu Zhao, Wei Zhang, Guohan Gao, Bo Zeng, Changtao Wang and Xiangang Luo

State Key Laboratory of Optical Technologies on Nano-Fabrication and Micro-Engineering, Institute of Optics and Electronics, Chinese Academy of Sciences, P.O. Box 350, Chengdu 610209, China

*Correspondence and requests for materials should be addressed to X.G.L. (e-mail: lxg@ioe.ac.cn)

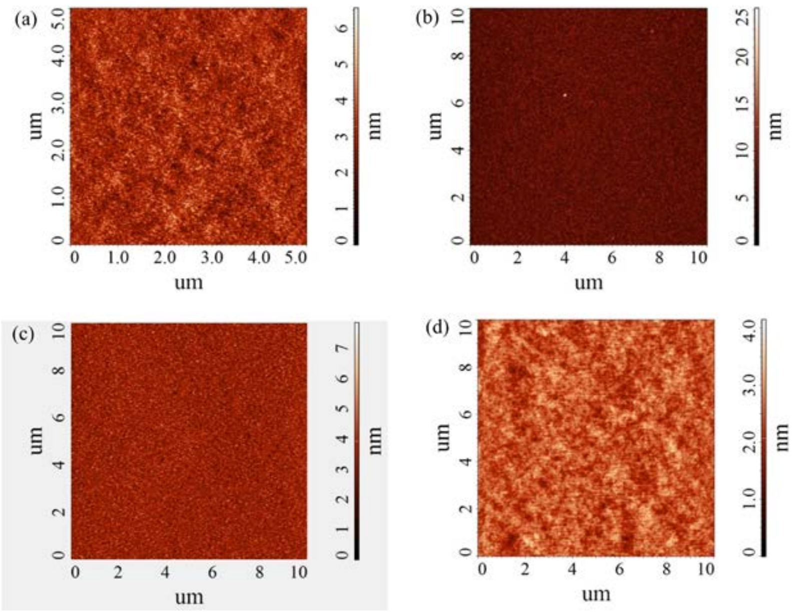

Figure S1 | Surface morphology of films measured by AFM. (a) RMS is 0.7nm for 20nm-thick SiO_2 film fabricated by SiO_2 sputter target; (b) RMS is 1.8nm for 20nm-thick A1 film fabricated by Al sputter target; (c) RMS is 0.9nm for 20nm-thick A1 film fabricated by alloyed Al sputter target with 3% Cu; (d) 50nm-thick PR with RMS 0.4nm.

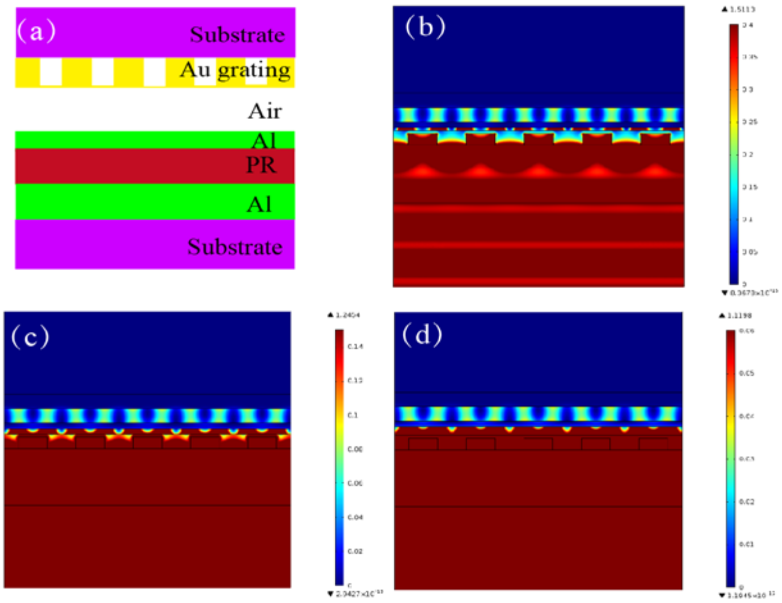

Figure S2 | (a) Schematic of odd SPP modes interference for air spacer structure. Cross section of normalized intensity distribution in logarithm scale inside the structure depicted in (a) with air distance 10nm for (b), 20nm for (c), and 30nm for (d).

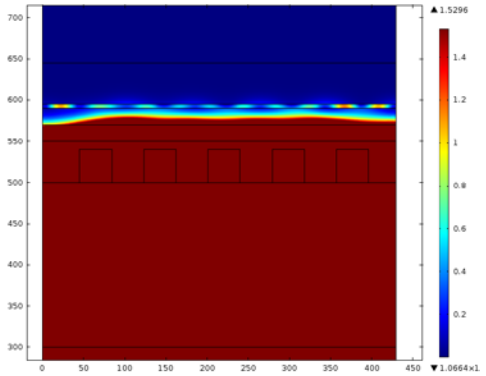

Figure S3 | For 5nm-thick PR, electric field intensity distribution in logarithm scale inside the odd SPP interference structure with mask pitch 78nm, and the other parameters are the same as those depicted in Fig. 1, and generate 19.5nm ($\sim\lambda/19$) half pitch interference patterns in PR layer, corresponding to the odd SPP modes at about $4.67k_0$
